# The Complexities Associated with Caring for Hospitalised Infants with Neonatal Abstinence Syndrome: The Perspectives of Nurses and Midwives

**DOI:** 10.3390/children8020152

**Published:** 2021-02-17

**Authors:** Jaylene Shannon, Stacy Blythe, Kath Peters

**Affiliations:** 1 Generalist Community Nursing, Mid North Coast Local Health District, Wauchope, NSW 2446, Australia; jaylene.shannon@health.nsw.gov.au; 2 School of Nursing and Midwifery, Western Sydney University, Penrith, NSW 2751, Australia; k.peters@westernsydney.edu.au

**Keywords:** neonatal abstinence syndrome, holistic care, nurses, midwives, NAS, NICU, special care nursery

## Abstract

The global incidence of Neonatal Abstinence Syndrome (NAS) has increased significantly in the last decade. Symptoms of NAS manifest from the central and autonomic nervous systems as well as the gastrointestinal system and vary in severity and duration. The clinical management of infants experiencing NAS is dependent on symptoms and may include both pharmacological and non-pharmacological measures. In cases where symptoms are severe, infants may be admitted to special care nurseries or neonatal intensive care units. Existing research on nurses’ involvement in caring for infants with NAS focuses on pharmacological and non-pharmacological interventions to treat physical symptoms associated with NAS. This research sought to add to the body of knowledge around NAS and conveys nurses’ and midwives’ experiences of delivering care for infants with NAS. Semi-structured interviews were held with nine nurses/midwives. Interviews were audio-recorded, transcribed verbatim and thematically analysed. Five themes emerged from the data. These themes are: Complex care needs; Prioritising physiological care; Experiencing compassion fatigue; Lacking continuity of care; and Stigma. The findings demonstrated the complex nature of care provision for infants with NAS. Competing priorities and the stigmatising nature of NAS threaten optimal care being delivered to these vulnerable infants and their parents.

## 1. Introduction

Neonatal Abstinence Syndrome is the presentation of withdrawal symptoms that 55–94% of all infants exposed to addictive substances during gestation will experience after birth [1]. The global incidence of NAS has increased significantly in the last decade [2]. In Australia, the prevalence of NAS is 42.2 per 10,000 live births [3]. Australian data estimate illicit drug use during pregnancy to be approximately 8% [4], compared to US survey reported data of 4% of pregnant women self-reporting illicit drug use during pregnancy [5]. This is compared to UK urban research, which found 15% of samples from pregnant women tested positive to illicit substances [6]. However, NAS can also occur from the use of prescription medications during pregnancy. An American study revealed 6% of pregnant women consumed prescription opioid medications for more than a month during pregnancy [7]. Another American study also revealed that approximately 1.8% of pregnant women use antidepressants and 3% use benzodiazepines to control depression and anxiety disorders [8]. Prior to 1970, NAS would occur secondary to the use of morphine or heroin abuse. Today, NAS may present secondary to the use of a variety of licit and illicit substances, including, morphine, methadone, heroin, buprenorphine, prescription opioids, antidepressants, anxiolytics and other substances [2].

Symptoms of NAS manifest from the central and autonomic nervous systems as well as the gastrointestinal system and vary in severity and duration [1]. Respiratory and metabolic symptoms include fever, mottling, sweating, tachypnea and nasal stuffiness. Gastrointestinal manifestations include projectile vomiting, watery stools, poor feeding and excessive sucking. Central nervous system manifestations include hyperirritability, high-pitched crying, tremors, sleep disturbances, myoclonic jerks and seizures. The onset of symptoms ranges from 24–72 h after birth and can persist for as little as 2 days or as long as 30 or more days [2].

Babies born to mothers who have used substances during pregnancy are also more likely to be pre-term, small for gestational age, experience birth complications, require pharmacological treatment, be admitted to the special care nursery (SCN) or neonatal intensive care unit (NICU), and experience a longer hospital admission and length-of-stay [4]. The current literature on nurses’ and midwives’ involvement in caring for NAS infants is largely based on pharmacological and non-pharmacological interventions to treat physical symptom management of NAS. There is comparatively less research investigating the experiences of NICU and SCN nurses/midwives in caring for these infants. Research that has explored nurses’/midwives’ experiences, reported substantial challenges associated with providing specialised care for the infant with NAS as well as working with their families within a family centred model of care [9,10,11]. This paper highlights some of the complexities inherent in the care of hospitalised infants with NAS from the perspectives of nurses and midwives.

## 2. Materials and Methods

### 2.1. Design

This paper presents one aspect of findings from a qualitative study. Therefore, a more comprehensive description of the methods for this study are presented in another paper in this special edition. A qualitative approach underpinned by Naturalistic Inquiry as described by Miles and Huberman was used to collect narrative data from nurses and midwives who had experienced caring for infants with NAS [12].

### 2.2. Recruitment and Data Collection

Prior to recruitment, approval was gained from the Western Sydney University Human Research Ethics Committee [Approval No: 12098]. Australian nurses and midwives who had experience in caring for infants with NAS were invited to participate in the study via a recruitment poster distributed to acquaintances of the research team and via snowball sampling.

Data were collected via semi-structured interviews. Participants were given the choice of attending a face-to-face interview or completing the interview online or via telephone. Prior to interviews, all participants provided written, informed consent. During interviews, participants were asked a series of open-ended questions and encouraged to share their experiences of providing care to infants with NAS in the NICU or SCN. Interviews were audio-recorded and transcribed verbatim by an external transcription company. Transcripts were de-identified to ensure participant confidentiality. Excerpts of transcripts portrayed throughout the findings were allocated identifiers (e.g., RN#) rather than pseudonyms.

### 2.3. Data Analysis

The accuracy of the transcriptions was ensured by listening to the audio-recordings while reading the transcripts. This also served to ensure familiarity with the data. Once checked for accuracy, participants’ transcripts were read several more times to gain a more in-depth understanding of participants’ narratives. Transcripts were then coded and grouped together into categories. These categories were discussed by all authors and further developed into themes which were finalised once consensus was reached.

## 3. Results

In total, nine nurses and midwives participated in the study. Analysis revealed three major themes, each with multiple subthemes. The first two themes—(1) Facilitating the attachment relationship; and (2) Barriers to promoting attachment—found that nurses and midwives understood the importance of infants developing a healthy attachment to their primary care-giver (usually the mother), but encountered multiple barriers to facilitating such attachment. Healthy attachment is the foundation of infant mental health. Attachment theory, as defined by Bowlby (1969), asserts infancy to be a period of rapid neurological development that is experientially and environmentally influenced [13]. Simply put, infants who experience sensitive, responsive caregivers develop neurological pathways enabling healthy psychosocial and emotional development [14]. Without this attachment, infants are left predisposed to complex developmental mental health disorders [15,16]. The findings describing the predisposition of hospitalised infants with NAS to develop *un*healthy attachments are reported in a separate article within this special issue.

This paper presents content from the final major theme: (3) Complexities associated with care for hospitalised infants with NAS. The sub-themes below (Table 1) highlight the complexities associated with the care of a baby with NAS. They explore the difficulties that nurses and midwives are faced with when an infant is admitted to the NICU/SCN due to the unique nature of NAS and how the disease differs so profoundly from other cases in the NICU and SCN units. The first sub-theme, “Complex care needs,” discusses nurses’ and midwives’ recognition of the need for highly experienced staff to care for infants experiencing NAS due to the complexity of the condition. The differing priorities of care for the infant experiencing NAS compared to other infants in the nursery is discussed in the sub-theme, “Prioritising physiological care.” The sub-theme “Experiencing compassion fatigue,” explores the impact that infants experiencing NAS can have on the job satisfaction and stress levels for nurses and midwives, and the sub-theme, “Lacking continuity of care,” explores the importance of continuity of care for infants experiencing NAS and how this may or may not occur while the infant is in the NICU/SCN. The final sub-theme, “Stigma”, highlights the stigmatising nature of NAS as a condition which potentially impacts on the care delivered to infants with NAS and their families.

### 3.1. Complex Care Needs—“The More Senior the Staff the Better off They Are Caring for the Babies”

All participants agreed that caring for infants experiencing NAS was complex. Additional time was required to manage symptoms effectively and support the family appropriately. However, participants were working within a system that did not facilitate adequate standards of care. Specifically, the nurse to patient ratio did not allow enough time for nurses/midwives to adequately provide comfort to an unsettled infant with NAS.

“It is often difficult though, if they’re in a one and three allocation, to always get to those patients in a timely manner.”

RN 6

“So if you’ve got four babies to look after in a Level 2 area, it’s very difficult to care for a baby who’s unsettled because of a NAS, because they’re withdrawing. Plus you’ve got a complex family to deal with, so you need time.”

RN 8

Participants recognised that infants with NAS required a lot of nursing time due to their need for additional comfort.

“A NAS baby is just really having a really rough time in that transition from an intrauterine life to extra uterine and they just really need that extra comfort and that comes down to time.”

RN 9

It was expressed that meeting the needs of infants with NAS was highly demanding. The consequence of not having enough time to promptly meet the needs of an infant with NAS resulted in the escalation of symptoms for which they required increased doses of medications.

“It’s just, it’s mainly they can be really, really challenging and I think that they don’t need to be as riled up as they are. In the sense that with a NAS baby if you can meet their needs quick enough they won’t be able to get into that state where they reach that inconsolable point and then their NAS scores are going really high and they need more medication.”

RN 9

Participants acknowledged the complexities of caring for infants with NAS in the NICU/SCN and conveyed that the allocation of senior nurses or midwives to care for was considered best practice due to the specialised knowledge required.

“We find here that the more senior the staff the better off they are caring for the babies, because they’ve had the most experience. So the more experience you have the easier it is to—you know how to swaddle them, feed them, not snack feed them or overfeed them.”

RN 8

“Often they can be—you know if you have a junior staff member looking after them they can be quite overfed, because every time they cry they get fed, that type of thing.”

RN 8

While it was agreed that infants with NAS required a lot of time and work from experienced nurses/midwives, participants reported that due to inadequate staffing, babies of a higher physiological acuity took precedence for allocation of experienced nurses/midwives over infants with NAS:

“Short of staff is always the ongoing problem for every hospital. Those experienced nurses usually get allocated to look after much sicker babies.”

RN 5

### 3.2. Prioritising Physiological Care—“Put the Baby down, We Don’t Have Time for That”

Participants understood comfort measures and symptom management were fundamental in the care of infants experiencing NAS, and therefore took priority.

“…realistically with a NAS baby they need, I find they need comfort more than anything else,”

RN 9

“I think symptom management—would be the biggest priority. I think most of the focus is on the baby so I think it’s reducing their—doing non-pharmacological and pharmacological interventions to reduce the symptoms of it, which I would say would be the priority of it. I wouldn’t say that the parents would be a priority. I would definitely say that the infant is the priority of the care.”

RN 7

Despite acknowledging comfort was integral to their care, due to health system constraints, meeting the physiological needs of the infant with NAS was the focus of their treatment.

“The priorities are to have—we prioritise their treatment, is to probably keep them stable, comfortable, not distressed, well fed, you know care for their skin, because you know they’re really prone to diarrhoea and stuff like that, so you really have to be on the ball with that and instigate—like use bottom creams and things like that if you start to notice nappy rashes. Feed them properly, don’t over-feed them. Handle them quietly, firmly, but gently and you try to not do anything to them that will stress them. Don’t have them in loud places. Be quiet around them. Don’t tiptoe, but be quiet around them.”

RN 8

Time constraints due to high nurse-patient ratios and high patient acuity, meant that the physiological needs of other infants in the NICU/SCN were often prioritised over the psychological comfort needs of infants experiencing NAS.

“I think if it’s predominantly the carers being nurses, I think we just do our best. I mean our attention is divided. It’s not the only infant you’ll have in your load for the day, so I guess again about prioritising infants’ needs, that—as much of—their needs are met around basic care, feeding, medication. Someone’s upset—oh look, I can’t attend to that baby right now.”

RN 2

The reason for prioritising physical care over emotional care of the newborn was based on lack of time experienced by nurses and midwives and also the perception that nurses and midwives were there to provide physiological care. This led to practices where it was only acceptable for nurses and midwives to fulfil comfort requirements of infants with NAS when the time pressures of the job allowed for this to happen.

“Because a lot of other midwives, older midwives or midwives that have been there a while, maybe they’re a bit burned out. They just say, “oh put the baby down, we don’t have time for that”. You know, go and—sorry the baby has to cry, that’s not our problem.”

RN 4

“Yeah, if it’s slow I’ll cuddle the baby. I’ll bring the baby—in the maternity ward I’ll bring the baby out to the nurses’ station with me, if I have the time while I’m doing the notes.”

RN 4

Participants recognised the problematic nature of prioritising physical over psychological care for these infants and acknowledged that nursing and midwifery staff currently working with infants with NAS were ill-equipped to cater to their mental health needs.

“I just think the best place to be would be mother and baby units where their mental health can be looked after a bit better, because we don’t have specific training in mental health disorders or the psych med or anything like that.”

RN 8

Involvement of parents in the care of infants with NAS only became a priority when it was recognised as a strategy for increasing the chances of early discharge.

“I think for me my goal was to get the baby to a point where they were settled and they were able to go home. I had to get through my nursing tasks, but I was thinking that the outcome was the quicker we can get the baby through this, get them attached to the mother, I think that that allowed them to get home quicker.”

RN 1

The consequence of not involving parents in the care of the infant with NAS was that frequently these infants did not bond with or attach to anyone during their time in the NICU/SCN.

“If the baby has nobody, they’re just lying there all day with nobody to cuddle besides us. Because like I said, we don’t exactly have the time, even though I’d love to just do that.”

RN 4

### 3.3. Experiencing Compassion Fatigue—“Oh God I’m Glad I’m Going to Go Home”

The challenging nature of caring for infants with NAS and their families resulted in increased stress being experienced by participants. Participants’ narratives indicated that existing models of care did not adequately support nurses/midwives in providing care for these infants. This is evident in the following dialogue when the participant reported her feelings of relief at the completion of her shift after caring for an infant experiencing NAS:

“We’re looking after them and after eight hours if you have a baby that’s really withdrawing, you’re going oh god I’m glad I’m going to go home.”

RN 7

Participants reported feelings of distress on behalf of the infant when they witnessed their withdrawal symptoms, and this made their job difficult.

“I mean it’s—it’s hard to see them when they’re so distressed. It’s probably one of the hardest parts of my job, to see a baby that’s—you just can’t settle.”

RN 3

One participant described the distress she felt on behalf of the infant when she realised the infant was not receiving the benefits of having a constant caregiver to provide comfort to the infant:

“Whereas he just would sometimes cry and cry, and nobody could get to him, and he would just give up. He would just have this sort of—sometimes he’d just be there with this sort of look. I don’t know. Maybe it was in my head, but it just seemed like he had given up on—it was like crying is not working, so I’m just going to lie here, you know? So yeah, there definitely seemed to be a difference.”

RN 4

Keeping the infant with NAS comfortable and settled was reported to be highly time consuming for the participants. The increased time required to care for infants with NAS, and the intensity of the symptoms that presented during withdrawal, resulted in increased stress experiences for nurses and midwives. Participants acknowledged the importance of continuity of care for these infants, but reported that nurses and midwives can quickly become fatigued if they have to continue to care for the infant with NAS over continuous shifts.

“Dare I say it, the nursing staff as well sometimes get quite stressed because the babies can be quite intense to look after. In saying that, providing continuity of care is great, but if you cop a couple of shifts with a particular baby that’s really losing it, the nurses can get really burnt out.”

RN 8

### 3.4. Lacking Continuity of Care—“NAS Baby in a Nursery Usually Gets Allocated to Agency Nurse or Grad Nurse”

Participants recognised the importance of continuity of care for keeping infants with NAS settled and comfortable. They recognised that familiarity was key to being able to successfully soothe the infant with NAS and therefore continuity of care was essential for these infants.

“You find that these babies get really unsettled with different staff members who have not looked after them before, and continuity of care is probably the crux of that as well.”

RN 8

“If they’re on a full withdrawal and they’re screaming their lungs out and they’re shaking and they’re really hard to calm if you don’t feel an attachment to the baby, or if the baby isn’t responsive to you, it’s just not going to get anywhere very, very quickly.”

RN 9

Despite the recognition that infants experiencing NAS require continuity of care, participants highlighted that due to existing models of care that preference physiological over psychological care, these infants were often allocated to agency staff, casual staff, or less experienced staff members, and this impacted on the quality of care delivered and resulted in a lack of continuity of care.

“NAS baby in a nursery usually gets allocated to agency nurse or grad nurse or less experienced nurse. The end with this NAS baby usually doesn’t receive very good grounded care because you do need special training to look after this kind of baby.”

RN 5

This lack of continuity of care often negatively impacted on the establishment of a relationship with both the infants experiencing NAS and with their caregivers. It was acknowledged that without continuity of care, parents of the infant with NAS could not establish relationships with nurses and midwives and therefore, care delivered to parents and the infant was difficult.

“I suppose one part—and the nursery is particularly—and the lower ends of the nursery—that often they get agency staff or people coming in and out. I think it’s hard for the parents because they can’t form that relationship with the staff, but also too that the babies don’t form that relationship either with staff.”

RN 7

The long-term implications of a lack of continuity of care was also acknowledged by participants. The following dialogue expresses the importance of continuity of staff members to ensure that family-centred care is provided in order to ensure that the infant will be going home to a supportive environment, and that the family has an adequate support network.

“…and if this baby doesn’t get enough care probably get overlooked by staff and when the baby is discharged maybe the baby doesn’t end up with a very good follow up or the mum lost support and more problems could emerge after discharge”

RN 5

### 3.5. Stigma—“There’s a Culture of Negative Feeling towards the Family”

Participants identified that the notion of the condition came with a lot of stigma and therefore became a taboo topic.

“I mean it’s never going to be a popular sort of disease process sort of thing. It’s one of those difficult to talk about. There’s lots of things around privacy and confidentiality and you can’t identify the infants. So it’s not something that’s talked about a whole lot.”

RN 2

It was identified that the condition of NAS was a negative topic that often evoked feelings of alarm for the participants when a baby would experience NAS due to the mother having a history of drug addiction. Participants felt that it was often obvious to other parents of infants in the NICU/SCN, and participants felt sympathy for the other parents because they had to witness the withdrawal symptoms of the infant with NAS, as well as the behaviours of the parents with a history of substance use.

“In a way, I think it can be really, you know we always find it quite a negative subject don’t we, as nurses? Everyone will say, because we house them in our transition nursery, everyone will say oh my god there’s four NAS babies up there or something like that, and you know sometimes when they’re really noisy I feel for other parents, because they certainly know what’s going on as well, don’t they? You know they know that that kid’s withdrawing from drugs.”

RN 8

“I always feel sorry for parents that have never been involved in that sort of situation and they see these sort of parents that come in and visit their babies and can be loud and aggressive and they have to see us deal with it.”

RN 8

Participants acknowledged that the stigma of the condition was embedded into the culture of care for infants with NAS and led to the infant’s parents being blamed and deemed undesirable.

“I think somehow there’s a culture, we’ve got a NAS baby, must come from a mummy not control herself very well and there’s a culture of negative feeling towards the family behind this NAS baby.”

RN 5

Participants described negative feelings that they experienced towards a mother of an infant experiencing NAS and how difficult it was to refrain from being judgmental of the social conditions to which infants with NAS were exposed.

“I must admit my personal experience when I first started looking after NAS babies I use to find myself feeling quite angry at the parents.”

RN 9

“Part of me thinks well, I’m sorry but you deserve it, but that’s not really me.”

RN 3

The stigma attached to the condition and the parents of the infant with NAS, also extended to the culture of care for infants themselves.

“For the past three to five years the care still almost the same—NAS baby always put into the corner.”

RN 5

Participants often felt that parents of infants with NAS obstructed the nurses’ or midwives’ ability to provide nursing/midwifery care to the infant.

“I think a lot of neonatal nurses see NAS parents as more of a hindrance than a help.”

RN 7

“They are really good at over-handling the baby and unsettling them again. They often undo all the work that you’ve already done.”

RN 8

The stigmatisation of parents and NAS as a condition served to discourage nurses and midwives from providing family centred care. Participants reflected that often nurses and midwives thought that working with families was pointless as they believed that the parents would need to relinquish custody of the child.

“And I don’t think any of the nurses and midwives do see the importance of that mother/infant bond. I think there is a proportion who goes oh it doesn’t matter; they’re going to get taken off them anyway.”

RN7

Despite this stigmatisation, participants recognised the difficulties for parents to be in an environment where they were being judged by health care providers.

“So even when they are trying to do the right thing they’re always being judged based on other experiences that nurses and midwives have had, which I think is sad because it makes them stop trying. You’re not supporting them and going good on you, you’re doing so well, rather than being always suspicious of them.”

RN 7

## 4. Discussion

This paper has provided new insights into the complexities associated with the provision of nursing care to hospitalised infants with NAS and their families. Although not generalizable, the findings reported here resonate with extant literature. Participants’ difficulties providing adequate, consistent care to hospitalised infants with NAS and their families have been reported for more than a decade [9,10,11,17], demonstrating the persistence of these issues.

Stigma and negative attitudes of health professionals are known to impact a mother’s willingness to access health-related services [18]. Howard et al. (2017) interviewed parents of infants with NAS and revealed that some of the reasons for maternal absence from the NICU/SCN included their experiences of both stigma and feelings of guilt [19]. Parents of infants admitted to a NICU or SCN often endure extreme stress and confusion about the wellbeing of their infant [20]. For the parent of an infant with NAS, this stress may be exacerbated by fear of judgment from health professionals and subsequent child protection involvement [19]. As a result, parents may avoid the NICU/SCN in order to minimise attention from staff. Parental absence from the NICU/SCN served to increase nursing workload and perpetuate parental stigmatisation as nurses were subsequently needed to offer soothing and comfort to the infants. Such activities are generally seen as parental responsibilities and not part of nursing care.

Nurses and midwives are in a unique position to provide support, education and encouragement to the family with an infant experiencing NAS [21]. Furthermore, nurses in the NICU generally strive to work with families of infants in their care, acknowledging the benefits of family centred care for optimal health outcomes [22]. Integral to family-centred care are respectful and trusting relationships between parents and health care providers. However, in the current study it was noted that the stigma of the condition affected nurses’ and midwives’ willingness to interact with parents, and negative and judgmental attitudes towards the parents was prominent within the NICU/SCN settings. A lack of empathy, understanding and knowledge of the history and background of the parents and their substance use, was recognised as contributing factors for these attitudes. Participants noted that parents of infants with NAS were expecting to be judged by nursing staff and experienced feelings of guilt and shame for their infant’s condition. This finding is consistent with other qualitative studies of nurses’ experiences with NAS families [9,10,11].

A finding that has not yet been noted in the literature, but was revealed in the current study, was that the stigmatisation of parents extended towards stigmatisation of the infant with NAS. It was identified that infants experiencing NAS impacted nurses’ and midwives’ ability to meet job demands due to the infants’ constant need to be soothed and comforted. This increased demand led to a lack of continuity of care for the infant with NAS as nurses and midwives experienced a reluctance to care for infants due to their increased needs as well as having to negotiate the complex nature of the family. As a result, it was noted that infants with NAS would be allocated to agency staff resulting in a lack of continuity of care. This is of concern, as effective continuity of care has been known to decrease length of stay for infants in the NICU [23,24]. In addition to potentially increasing the length of stay for infants with NAS, lack of continuity of care, inadequate staffing levels and poor communication have been identified as causes of moral distress among nurses in the NICU setting [25].

The nurses/midwives in the current study believed that continuity of care for infants with NAS was important to establish rapport with the infant’s family, and to dissolve judgement by developing empathy. However, the increased needs of the infant with NAS and the competing job demands of being a nurse/midwife in the NICU/SCN, contributed to increased stress experienced by nurses/midwives. Subsequently, continuity of care was sacrificed in order to prevent and/or minimise the demands on staff. This disengagement from patients is a common sign of compassion fatigue, a unique form of burn out that relates to the continual, relational interactions with individuals and families needing help [26]. Compassion fatigue is not uncommon among nurses and can negatively impact patient care, workplace environment, and productivity [26,27]. Findings from this current study resonate with previous research that acknowledged that nurses caring for infants with NAS are at significant risk of experiencing compassion fatigue [27].

When nurses/midwives experience an increased demand on their service which may exceed their available time, decisions about which tasks take precedence over others and the order in which these tasks need to be carried out, must be established [28]. In the current study, participants described allocations of patients of up to four infants in the NICU/SCN per nurse/midwife, with each infant requiring care and attention, not just as a newborn baby, but also due to complex health conditions. Nurses/midwives described the dilemmas they faced when they had to prioritise the care of other infants in the nursery over tending to infants that were crying. Prioritising care to infants with greater physiological needs resonates with findings from previous research, where NICU nurses considered babies with NAS as less ‘sick’, and preferred to focus on the high-tech aspects of their work rather than provide comfort to a baby with NAS [11].

The decisions nurses/midwives make about patient care priorities can have a substantial and lasting impact on the patient’s outcome. However, determining priorities can be complex and confusing for nurses in various patient situations due to differing priority needs of patients. Often, physiological needs of patients can also be prioritised over psychological ones, due to time pressures in the hospital environment. Coinciding with the high-pressure environment of the NICU/SCN, nurses and midwives can experience the complexity of providing care to the infant and family affected by NAS. As a result, stigma surrounding the condition can impact on nurses’ and midwives’ willingness to engage in interactions with the family and nursing/midwifery care of the infant with NAS [9]. These barriers interfere with nurses’ and midwives’ ability to provide holistic care to the infant with NAS and their families.

Limitations of this study include the small sample size. Despite this small sample size, interviews gleaned rich narrative data from participants which provided new insights into the challenges experienced when delivering care to an infant with NAS. A further limitation was the inclusion of Australian nurses and midwives only, which may limit the transferability of the findings to other countries.

Our findings showed that compassion fatigue hindered the nurses’/midwives’ ability to provide optimum care to infants with NAS. To ensure the delivery of care to these infants and their families is both equitable and appropriate, nurses/midwives need to be adequately supported by health care systems in terms of the provision of lower patient-staff ratios, focused education, and a staffing skill mix conducive to alternative models of care [29,30]. Introducing different models of care that are baby-centred, focused on non-pharmacological care, and consider the mother as essential to the care of the infant, may be beneficial for infants with NAS and their families [31]. Further research is required that aims to implement and evaluate the effectiveness of such models of care in improving patient outcomes as well as nurses’/midwives’ experiences of providing care to infants with NAS.

## 5. Conclusions

There are multiple complexities associated with the provision of effective nursing care to infants with NAS and their families. Stigmatisation of substance using parents often resulted in their absence from the bedside, subsequently increasing nurses’ workloads as they attempted to comfort and soothe infants in withdrawal. Nurses prioritisation of physiological care aspects meant infants with NAS were often left crying for long periods of time while infants requiring specialised nursing care were seen first. The increased workload and complex family circumstances associated with caring for infants with NAS predisposed nurses to compassion fatigue, resulting in discontinuity of care for the infants and their families.

## Figures and Tables

**Table 1:** Summary of sub-themes.

Sub-Themes
Complex care needs: “The more senior the staff the better off they are caring for the babies” Prioritising physiological care: “Put the baby down, we don’t have time for that” Experiencing compassion fatigue: “Oh God I’m glad I’m going to go home” Lacking continuity of care: “NAS baby in a nursery usually gets allocated to agency nurse or grad nurse”
Stigma: “There’s a culture of negative feeling towards the family”

## Data Availability

The data presented in this study are available on request from the corresponding author. The data are not publicly available due to privacy restrictions.
